# Macromolecular Interactions of Disordered Proteins

**DOI:** 10.3390/ijms21020504

**Published:** 2020-01-13

**Authors:** István Simon

**Affiliations:** Institute of Enzymology, RCNS, Lorand Eotvos Research Network, Center of Excellence of the Hungarian Academy of Sciences, Magyar Tudósok krt. 2., H-1117 Budapest, Hungary; simon.istvan@ttk.mta.hu

Proteins are social beings. Especially disordered proteins like company, they hardly act without interacting with another macromolecule. In most known cases, these other macromolecules are other proteins, sometimes nucleic acids and very seldom something else. Disordered proteins are rather newcomers in protein science. The first papers on these proteins came out in the fourth quarter of the last century. What is more, they were hardly recognized before the great paper of Wright and Dyson published in J. Mol. Biol. in 1999 [1]. By now, it is well known that a large portion of all existing proteins are intrinsically disordered under physiological conditions. They perform vital roles in many living cells. For more than a decade, it was generally thought that disordered proteins or disorder parts of partially disordered proteins have different amino acid composition than folded proteins have and various prediction methods were developed based on this principle. Dosztanyi et al. [2,3] provided a physical background of a disorder prediction methods (IUPred) by estimating the lowest value of the sum of the pairwise interaction energies between residues from the amino acid sequences without considering structural information. This calculated energy per residue value for globular proteins was well separated from the ones calculated for disordered (unstructured) proteins know at that time. This principle of pair energy estimation applied in IUPred also worked well, when the method ANCHOR [4,5] was developed to predict binding site within disordered parts of protein by which the disordered protein bounds to a folded one and its structure is formed upon binding. Those segments of the disordered proteins are identified as binding site where in amino acids, considering together with the average composition of folded protein, exhibit low enough pairwise interaction energy to be stable. Recently however shreds of evidence were accumulated about the existence of a different type of disordered proteins [6]. It turned out that some disordered proteins can undergo coupled folding and binding without the involvement of an already folded protein, but by intra-acting with disordered proteins. This second protein can be the same as the first one (formation of homodimers) or can be different (formation of heterodimers. They can also form higher order oligomers. These proteins which can stabilize their structure via “mutual synergistic folding” have residue compositions similar to that of the folded globular water-soluble proteins. Their residue compositions are different from the composition of the traditional disordered proteins, which can only be stabilized on the surface of an already stabilized macromolecule, in most cases on the surface of a folded protein. These traditional disordered proteins can be named as “coupled folding and binding” protein. Recently the “mutual synergistic folding” proteins were collected in a database MFIB [7], the “coupled folding and binding” proteins were collected in a database DIBS [8] and the structural and functional properties of these two types of protein were compared [9]. Beside the large variation of protein-protein interactions, in the past decade, more and more examples are found, where disordered proteins interact with non-protein macromolecules in various forms [10]. There is also a very new phenomenon when proteins, including disordered ones, are involved in phase separation, which can be a weak but functionally important macromolecular interaction [11].

Paper of two merged special issues on the same topic: “Functionally relevant macromolecular interactions of disordered proteins” are summarized and listed in the order of their online publication date. Research, review and concept papers listed separately, starting with the oldest one in this Editorial.

## 1. Research

In the first paper of this series, a study on the effect of acetylation on the phase separation tendency of Tau protein was reported by Ferreon et al. It is well known that intrinsically disordered protein Tau is involved in Alzheimer’s disease. Recently it was shown that Tau is capable of undergoing liquid-liquid phase separation, which involves weak protein-protein interactions and it is considered as an initiation of Tau aggregation observed in Alzheimer’s disease. In this work, it was shown that acetylation disfavors phase separation and aggregation of Tau, therefore, acetylation prevents the toxic effects of liquid-liquid phase separation dependent aggregation [12].

Srivastava et al. tried to decipher RNA-recognition patterns of IDPs in the next paper. They analyzed the protein-RNA complexes which undergo disordered to ordered transition (DOT) during binding. The DOT region is small and positively charged, like the binding sites in globular proteins. However, for DOTs of IDP have significantly higher exposure to the water, than their counterpart in structured protein. These findings can help to develop tools for identifying DOT regions in RNA binding proteins [13].

Contreras et al. studied a Protein (LrtA protein of Synechocystis sp. PCC 6803) which is oligomeric and folded in solution, but the single-chain is only folded and stable in their N terminal half of the polypeptide (residues 1–100) while the other half (101–197) is very unstable and rather disordered with chameleonic sequence properties. While disordered protein which undergoes mutual synergistic folding upon binding to each other, this is a rather rear case, when it happens only with half of the protein. The other half remains folded before and after self-association [14].

The origin of the thermal stability of eukaryotic proteins was studied and compared with that of thermophilic and mesophilic proteins of prokaryotes by Alvarez-Ponce et al. The eukaryotic model system was *Arabidopsis thaliana* at 22 and 37 °C, and they compare both the amino acid compositions and levels of intrinsic disorder of heat-induced and heat-repressed proteins. Heat-induced proteins are enriched in intrinsically disordered regions and depleted in hydrophobic amino acids in contrast to thermophile prokaryotic proteins [15].

A decision-tree based meta server to predict disordered parts of proteins and their residues involved in binding motifs has been developed by Zhao and Xue. The meta server is based on four predictors: DisEMBL, IUPred, VSL2, and ESpritz. The meta server provides higher accuracy than each of these independent predictors [16].

Arvidsson and Wright applied a protein disorder approach characterizing differentially expressed genes analyzing cell adhesion regulated gene expression in lymphoma cells. They checked if predicted protein disorder was differentially associated with proteins encoded by differentially regulated genes in lymphoma cells. Intrinsic disorder protein properties were extracted from the Database of Disordered Protein Prediction (D^2^P^2^). They concluded that down-regulated genes in stromal cell-adherent lymphoma cells encode proteins that are characterized by elevated levels of disorder [17].

The co-evolution of IDPs and folded partner proteins was studied by checking their evolutionary couplings. Pancsa et al. pointed that due to the lack of strict structural constraints, IDPs undergo faster evolutionary changes than folded proteins, which makes the reliable identification and alignment of IDP homologs difficult. They demonstrated that partner binding imposes constraints on IDP sequences that manifest in detectable inter-protein evolutionary couplings. It brings hope that IDP–partner interactions could soon be successfully dissected through residue co-variation analysis [18].

A principal part of the physical bases of disordered proteins involved in mutual synergetic folding in homodimers has been uncovered by Magyar et al. The authors concluded that homodimer proteins have a larger solvent-accessible main-chain surface area on the contact surface of the subunits, when compared to globular homodimer proteins. The main driving force of the dimerization is the mutual shielding of the water-accessible backbones and the formation of extra intermolecular interactions [19].

Szabó et al. reported their finding that disordered parts of Mixed Lineage Leukemia 4 (MLL4) protein are capable of RNA binding. They explored the RNA binding capability of two; uncharacterized regions of MLL4; with the aim of shedding light to the existence of possible regulatory lncRNA interactions of the protein They demonstrated that both regions; one that contains a predicted RNA binding sequence and one that does not, are capable of binding to different RNA constructs in vitro [20].

A method to characterize the hydration of proteins based on evaluating two-component wide-line ^1^H NMR signals is presented in the next paper. Tompa et al. also provided a description of key elements of the procedure conceived for the thermodynamic interpretation of such results. The results enable a quantitative description of the ratio of ordered and disordered parts of proteins, and the energy relations of protein–water bonds in aqueous solutions of the proteins [21].

Homma et al, studied the evolution rate structural domains (SDs) and intrinsically disordered regions (IDRs) of immune-related mammalian proteins. IDRs are generally subject to fewer constraints and evolve more rapidly than SDs. However, it turned out that for immune-related proteins in mammals, the evolution rates in SDs come close to those in IDRs [22].

Moosa, M.M. et al. applied direct single-molecule observation to study sequential DNA bending transitions by the SoxSox2 is a transcription factor which assumed to achieve its regulatory diversity via heterodimerization with partner transcription factors. However, single-molecule fluorescence spectroscopy suggests that Sox2 alone can modulate structural landscape of the DNA in a dosage-dependent manner [23].

In a paper which was a follow-up of the Contreras, L.M. et al. paper [14], Neira et al. reported a study on the structure of the C-terminal half (residues 102–191) of the LrtA protein of *Synechocystis* sp. PCC 6803 in separated form with various physical-chemical techniques. At physiological conditions isolated C-LrtA intervened in a self-association equilibrium, involving several oligomerization reactions. They concluded that C-LrtA was an oligomeric disordered protein [24].

Mishra et al. extended their one-bead-per-amino-acid model for intrinsically disordered proteins to account for phosphorylation in studying the effect of phosphorylation on nuclear pore complex selectivity. The simulations show that upon phosphorylation the transport rate of inert molecules increases, while that of nuclear transport receptors decreases. The models provide a molecular framework to explain how extensive phosphorylation decreases the selectivity of the nuclear pore complexes [25].

Walter et al. studied the hydrodynamic properties of the intrinsically disordered potyvirus genome-linked protein, VVPg), of the translation initiation factor, eIF4E, and of their binary complex (VPg)-eIF4E. N-terminal His tag decreased the conformational entropy of this intrinsically disordered region. A comparative study revealed the His tag contribution to the hydrodynamic behavior of proteins [26].

The role of intrinsically disordered linkers in the confinement of binding domains in enzyme actions was studied in the following paper. By statistical physical modeling Szabo et al. show that this arrangement results in processive systems, in which the linker ensures an optimized effective concentration around novel the binding site(s), favoring rebinding over full release of the polymeric partner. By analyzing 12 enzymes they suggest a unique type of entropic chain function of intrinsically disordered proteins, that may impart functional advantages on diverse enzymes in a variety of biological contexts [27].

Machulin et al. studied the contribution of repeats in ribosomal S1 proteins into the tendency for intrinsic disorder and flexibility within and between structural domains for all available UniProt S1 sequences. Using charge–hydrophobicity plot cumulative distribution function (CH-CDF) analysis they classified 53% of S1 proteins as ordered proteins, the remaining proteins were related to molten globule state. According to the FoldUnfold and IsUnstruct programs, relatively short flexible or disordered regions are predominant in the multi-domain proteins. Their results suggest that the ratio of flexibility in the separate domains is related to their roles in the activity and functionality of S1 [28].

The decrease of disorder level of p53-DBD upon interacting with the anticancer protein Azurin by mean of Raman spectroscopy was monitored by Signorelli et al. This technique was found to be suitable to elucidate the structural properties of intrinsically disordered proteins and was applied to investigate the changes in both the structure and the conformational heterogeneity of the DNA-binding domain (DBD) belonging to the intrinsically disordered protein p53 upon its binding to Azurin, an electron-transfer anticancer protein from *Pseudomonas aeruginosa*. The results show an increase of the secondary structure content of DBD concomitantly with a decrease of its conformational heterogeneity upon its binding to Azurin [29].

Structural and functional properties of a capsid protein of dengue and related flavivirus. Dengue, West Nile and Zika have very similar viral particle with an outer lipid bilayer containing two viral proteins in the nucleocapsid core were studied by Faustino et al. Using dengue virus capsid protein as the main model, the protein size, thermal stability, and function with its structure/dynamics features were correlated. Their findings suggest that the capsid protein interaction with host lipid systems leads to minor allosteric changes that may modulate the specific binding of the protein to the viral RNA [30].

Chan-Yao-Chong, et al. investigated the early steps of actin recognition of Neural Wiskott–Aldrich Syndrome Protein (N-WASP) domain V. Using docking calculations and molecular dynamics simulations, their study shows that actin is first recognized by the N-WASP domain V regions which have the highest propensity to form transient *α* –helices. The WH2 motif consensus sequences “LKKV” subsequently binds to actin through large conformational changes of the disordered domain V [31].

Mentes et al.’s paper is the follow-up of the Magyar’s paper [19] of this collection. It reports the properties of heterodimer Mutual Synergistic Folding (MSF) proteins instead of homodimeric ones. The main driving force of the dimerization is the mutual shielding of the water-accessible backbones and the formation of extra intermolecular interactions just like in homodimers. However here shielding of the β-sheet backbones and the formation of a buried structural core along with the general strengthening of inter-subunit interactions together could be important factors [32].

Conformational ensembles of alpha-Synuclein were studied using single-molecule force spectroscopy and mass spectroscopy by Corti et al. This work applies single-molecule force spectroscopy to probe conformational properties of α-synuclein in solution and its conformational changes induced by ligand binding. This analysis provides support to the structural interpretation of charge-state distributions obtained by native mass spectrometry and helps defining the conformational components detected by single-molecule force spectroscopy [33].

The topic of the Mészáros et al. paper is closely related to the ones of the Mentes et al’s. paper [32] and the Magyar et al. paper [19]. The authors report the sequence and structure properties of protein complexes formed by disordered proteins via Mutual Synergistic Folding (MSF). A method is presented which differences in binding strength, subcellular localization, and regulation are encoded in the sequence and structural properties of proteins. It serves as a better representation of structures arising through this specific interaction mode [34].

Three Rett syndromes (RTT) treatment-related genes MECP2, CDKL5 and FOXG1 in silico by evolutionary classification and disordered region assessment were reported in this paper. Fahmi, M. et al. provided insight into the structural characteristics, evolution and interaction landscapes of those three proteins. They also reported the disordered structure properties and evolution of those proteins which may provide valuable information for the development of therapeutic strategies of RTT [35].

## 2. Review

Sánchez-López et al. report about the structural determinants of the N-terminus of the prion protein and the effect of binding copper ions in their review. They discuss the current knowledge of how mutations can impact the copper-binding properties of prion protein both in health and disease progression [36].

In their review paper Ciemny et al. first introduced the technique of Monte Carlo and other simulation for predicting possible structures of unstructured protein, protein-peptide complexes and unfolded states of globular proteins. They presented several case studies on various disordered proteins. They also proposed the use of the CABS coarse-grained model with Monte Carlo sampling scheme. They also show that CABS can be combined with the use of experimental data too [37].

The literature information on the alteration of disordered proteins in neurodegenerative and other diseases reported in this review. Martinelli et al. discussed how the misfolded proteins can be involved in Alzheimer’s, Parkinson’s and other diseases. The most common form of misfolding IDPs is the formation of neurotoxic amyloid plaques. The review discusses important special cases of beta-amyloid, alpha-synuclein, tau etc. They also show drug candidates for later use in to treatment of diseases caused by misfolded IDPs [38].

This is the first review from the Greb-Markiewicz lab in which Kolonko and Greb-Markiewicz summarized our current knowledge on helix-loop-helix/Per-ARNT-SIM (bHLH–PAS) proteins, considering their structures and intrinsic disorder nature based on NMR and X-ray analysis. Currently, all determined structures comprise only selected domains (bHLH and/or PAS), while parts of proteins, comprising their long C-termini, have not been structurally characterized yet since these regions appear to be intrinsically disordered. These intrinsically disordered parts contribute a lot to the flexibility and function of these proteins [39].

The second review from the same lab is the paper of Tarczewska and Greb-Markiewicz which is a follow-up publication of the review paper of Kolonko and Greb-Markiewicz [39], the currently available information on “The Significance of the Intrinsically Disordered Regions for the Function of the BHLH Transcription Factors” is reported. Their aim was to emphasize the significance of existing disordered regions within the helix–loop–helix (bHLH) transcription factors for their functionality [40].

Finally, in the last review paper of this collection, Owen and Shewmaker summarized our current knowledge on “The Role of Post-Translational Modification in the Phase Transition of Intrinsically Disordered Proteins”. They pointed that intrinsically disordered regions are critical to the liquid–liquid phase separation that facilitates specialized cellular functions and discuss how post-translational modifications of intrinsically disordered protein segments can regulate the molecular condensation of macromolecules into functional phase-separated complexes [41].

## 3. Concept

In his concept paper, Rikkerin, E.H.A. considers the “first line response” role of disordered protein in the protection against pathogens and disease. He presents several examples of how disorder and post-translational changes can play in the response of organisms to the stress of a changing environment. He proposes that some disordered proteins enable organisms to sense and react rapidly as the first line responds [42].
